# Serum uric acid level can predict asymptomatic brain metastasis at diagnosis in patients with small cell lung cancer

**DOI:** 10.1186/s43046-024-00235-1

**Published:** 2024-09-30

**Authors:** Gizem Agtas, Ali Alkan, Özgür Tanriverdi

**Affiliations:** 1 Faculty of Medicine, Mugla Sıtkı Koçman University, Muğla, Turkey; 2 Department of Medical Oncology and Oncological Clinical Research Center, Mugla Sıtkı Koçman University Faculty of Medicine, Muğla, Turkey

**Keywords:** Uric acid, Brain metastasis, Small cell lung cancer

## Abstract

**Background:**

The aim of this study was to determine the relationship between serum uric acid level at diagnosis and asymptomatic brain metastasis in patients with extensive-stage small cell lung cancer.

**Methods:**

A total of 69 patients with extensive-stage small cell lung cancer without symptomatic brain metastases, whose serum uric acid level was measured at the time of diagnosis, were included in this retrospective cross-sectional study. The patients were divided into two groups as those with and without asymptomatic brain metastases. The Mann–Whitney *U* test was used for comparison between groups, and Spearman’s correlation test was used for correlation analysis. The cut-off level of serum uric acid level was analyzed, and sensitivity, specificity, and accuracy rates were determined for brain metastasis. Independent factors affecting asymptomatic brain metastasis were determined by multivariate Cox regression analysis.

**Results:**

The median serum uric acid level of all patients was 6.9 mg/dL. Twenty-two percent of patients had asymptomatic brain metastases, and serum uric acid levels were significantly higher in these patients (*P* = 0.0014). The cut-off value for serum uric acid level was calculated as 6.2 mg/dL. The sensitivity, specificity, and accuracy of this value for brain metastasis were 84%, 76%, and 78%, respectively. High serum uric acid level was an independent risk factor for asymptomatic brain metastasis (OR 3.446 95% CI 1.337–5.480; *P* = 0.005).

**Conclusion:**

In conclusion, a serum uric acid level of 6.2 mg/dL and above at the time of diagnosis may predict asymptomatic brain metastasis in patients.

## Introduction

Small cell lung cancer (SCLC) accounts for approximately 15% of lung cancer, which is still one of the leading causes of cancer deaths today [1, 2]. In addition to being very common in smokers, the tendency for early distant metastasis and the susceptibility to acquired drug resistance are the most important clinical features of SCLC. Immunohistochemical staining shows expression of typical neuroendocrine epithelial markers, including synaptophysin, chromogranin A, and insulinoma-associated protein 1 (INSM1) [2, 3]. Almost half of the patients are diagnosed at an advanced stage. While the 5-year survival rate is 27% in patients with limited-stage disease, this rate is reported to be around 5% in metastatic disease [2, 3]. Although they are sensitive to chemotherapy and radiotherapy, this response may not last exceptionally long. In the extensive stage, the addition of the programmed death ligand 1 (PDL1) inhibitor atezolizumab to the standard chemotherapy regimen consisting of a platinum agent combined with etoposide increased median overall survival from 10.3 to 12.3 months [2–4].

Metastasis to the brain is most common in lung cancer and accounts for approximately 40–50% of all brain metastases. It is estimated that 10–20% of patients with small cell lung cancer will present with brain metastases at diagnosis, and up to 50–80% of patients will develop brain metastases at some point in their lives [2–4]. Currently, there is no biomarker that predicts the presence of brain metastasis and the time it takes from diagnosis to brain metastasis regardless of the cancer stage for asymptomatic patients.

Determining the predictive and prognostic values of biomarkers for several types of cancer is an active area of research. The ideal biomarker would need to be easily accessible and measurable, financially feasible, and widely used. Uric acid is one of the molecules that has been the subject of a limited number of studies reporting important results in many types of cancer in recent years, especially in terms of its prognostic value [5–14]. Uric acid is one of the end products of nucleotide metabolism. Xanthine and hypoxanthine are oxidized by xanthine oxidase and metabolized to uric acid. Uric acid has pro-inflammatory properties and is therefore thought to play a role in the development of cancer [5–7]. Due to this feature, it is thought that increased serum uric acid level (SUAL) may be associated with poor prognosis, especially in cases where the tumor burden is high. Although there are studies in the literature on the predictive value of brain metastasis in non-small cell lung cancer, there are limited studies on the predictive value of SUAL in SCLC, which is an aggressive tumor with a high proliferative index.

The aim of the study is to determine the predictive value of SUAL measured at the time of diagnosis for asymptomatic brain metastasis in patients with advanced SCLC.

## Patients and methods

The study is a cross-sectional study designed to retrospectively review the demographic and medical records of patients with extensive-stage SCLC without symptomatic brain metastases. For the study, patients who were treated at the Medical Oncology Department Faculty of Medicine of Mugla Sitki Kocman University between July 2011 and January 2023 were retrospectively evaluated.

The study was initiated after obtaining the approval (date: May 30, 2022, and protocol number: 220025) of the Scientific Research Ethics Committee of Mugla Sitki Kocman University.

SCLC patients who had SUAL among the tests requested during the first routine oncology examination after diagnosis were included in the study. However, patients who had symptomatic brain metastasis at the time of diagnosis, patients with two or more primary solid tumors or accompanying synchronous or metachronous hematologic cancer, patients who were diagnosed with gut disease, metabolic syndrome, clinical thyroid dysfunctions, and who had alcohol consumption history were excluded from the study.

The following were defined as study variables: (i) demographic characteristics: age at the time of diagnosis (years), gender (female/male), smoking habit (present/absent), and performance status according to Eastern Collaborative Oncology Group (ECOG PS); (ii) clinical characteristics: the site of metastasis at the time of diagnosis, the number of metastasis sites at the time of diagnosis (single site/multiple sites), recently status (alive/dead), date of brain metastasis detection (to determine the time it took from diagnosis to brain metastasis); (iii) laboratory characteristics: SUAL at the time of diagnosis (mg/dl). Since the tests requested from the patients were normal outpatient routine blood tests, blood samples were taken after 12 h of fasting. All tubes were centrifuged for 15 min until 30 min immediately after the blood sample was obtained. Then the analysis of the samples was measured by the automatic colorimetric enzymatic method. Normal SUAL was 7–8 mg/dL in men and 6 mg/dL in women.

### Statistical analysis

Data were expressed as median and minimum–maximum (min–max) range or median and interquartile range (25–75%). The distribution of variables was analyzed with the Kolmogorov–Smirnov test. An independent Student’s *t* test was used for normally distributed variables, and the Mann–Whitney U test was used for non-normally distributed variables. The cut-off value for SUAL was determined using the ROC curve. The sensitivity, specificity, and accuracy of this cut-off value were calculated for asymptomatic brain metastases at the time of diagnosis. The relationship between study variables was made by Spearman correlation analysis. Multivariate Cox regression analysis was performed to identify independent risk factors for brain metastases using models created according to univariate analysis results. Odds ratios (OR) and lower–upper 95% confidence interval (CI) values for independent factors were demonstrated. Statistical analysis was performed using the SPSS v.21.0 program. If the *P* value was less than 0.05, this value was considered statistically significant.

## Results

**Fig. 1:**
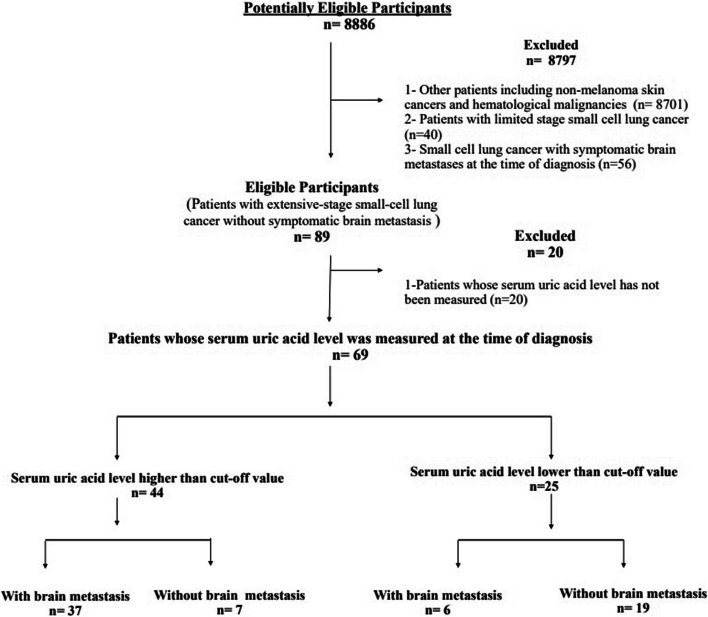
Study flow

A total of 69 out of 598 lung cancer patients met the study criteria and were analyzed for the study (Fig. 1). The median age (min–max range) of the patients was 54 years (48–71). Most of the patients were women (*n* = 37, 54%), smokers (*n* = 58, 84%), those with ECOG PS 2 (*n* = 48, 70%), and those with multiple metastatic sites (*n* = 45, 65%). The most common metastasis sites were the lungs (*n* = 30, 44%) and pleura (*n* = 28, 41%). The median SUAL calculated with the 25th and 75th interquartile percentile for all patients was 6.9 mg/dL (6.1 and 8.4, respectively) (Table 1).

**Table 1:** The demographic, clinical, and laboratory features of all patients included in the study as well as a comparison of these features according to the presence of brain metastasis at the time of diagnosis

Variables	All patients	Patients with asymptomatic brain metastasis	Patients without brain metastasis	*P* value^α^
***n* (%)**	**69**	**15 (22)**	**54 (78)**	
**Gender; *n* (%)**				
Female	37 (54)	8 (53)	29 (54)	0.307
Male	32 (46)	7 (47)	25 (46)	
**Age (year); median (min–max)**	54 (48–71)	57 (51–68)	53 (48–71)	0.116
**Smoking habits; *n* (%)**				
Present	58 (84)	12 (80)	46 (85)	0.245
Absent	11 (16)	3 (20)	8 (15)	
**ECOG PS**				
0	6 (8)	2 (13)	4 (7)	**0.037**
1	48 (70)	6 (40)	42 (78)	
2	15 (22)	7 (47)	8 (15)	
**Number of metastatic sites; *n* (%)**				
Single site	24 (35)	2 (13)	22 (41)	**0.029**
Multiple sites	45 (65)	13 (87)	32 (59)	
**Metastasis sites; *n* (%)**				
Lung	30 (44)	12 (80)	18 (33)	**0.009**
Pleura	28 (41)	12 (80)	16 (30)	
Liver	24 (35)	9 (60)	15 (28)	
Bone	24 (35)	9 (60)	15 (28)	
Adrenal gland	20 (29)	9 (60)	11 (20)	
Intraabdominal lymph nodes	12 (17)	9 (60)	3 (5)	
Supraclavicular/cervical lymph nodes	10 (16)	7 (47)	3 (5)	
Peritoneum	4 (6)	2 (13)	2 (4)	
**SUAL (mg/dl) median (Q1, Q3)**	6.9 (6.1, 8.4)	7.2 (6.8, 8.4)	6.6 (6.1, 7.2)	**0.0014**

*Abbreviations*: *Min* minimum, *Max* maximum, *ECOG* Eastern Collaborative Oncology Group, *PS* performance status, *Q1* 25% percentile, *Q3* 75% percentile, *SUAL* serum uric acid level

^α^Since the data did not show the normal distribution in the groups, the Mann–Whitney *U* non-parametric test was used to compare the two groups. If the *P*-value was less than 0.05, it was considered statistically significant. Statistically different data is indicated using bold font

Fifteen (22%) of the patients had asymptomatic brain metastasis at the time of diagnosis. When patients with brain metastases at the time of diagnosis were compared with those without brain metastases, it was determined that the PS of patients with brain metastases was worse (*P* = 0.037), and they had more multiple metastatic sites (*P* = 0.029). In addition, patients with brain metastases had significantly higher SUAL values at the time of diagnosis than those without brain metastases (*P* = 0.0014) (Table 1).

The median SUAL in the patients with and without brain metastasis was 7.2 mg/dL (6.8, 8.4) and 6.6 mg/dL (6.1, 7.2), respectively. SUAL was significantly higher in patients with brain metastasis (*P* = 0.0014) (Table 1). Then, all patients in the study were divided into two groups according to the SUAL cut-off value: high SUAL (*n* = 44) and low SUAL (*n* = 25) (Fig. 1). This stratification was used in correlation analysis as well as univariate and multivariate analyses.

When the target group was taken as patients with brain metastases, the area under the ROC curve was 67.4%. With Youden’s index, the cut-off value for SUAL was determined as 6.2 mg/dL. When analyzed according to the cut-off value; it has 83.78% (95% CI 69.93% to 93.36%) sensitivity, 76% (95% CI 54.87% to 90.60%) specificity, and 77.78% (95% CI 66.16% to 86.91%) accuracy rates for asymptomatic brain metastases if SUAL is 6.2 mg/dL and above at the time of diagnosis. The positive predictive value was 49.70% (95% CI 34.11% to 65.34%) and the negative predictive value was 94.42%. (95% CI 77.82% to 99.62%).

It was found that there was a moderate and positive correlation between the presence of asymptomatic brain metastases at the time of diagnosis and ECOG PS 2 (*r*_spearman_ = 0.401, *P* = 0.045), a moderate and positive correlation with bone (*r*_spearman_ = 0.456, *P* = 0.024), and supraclavicular/cervical lymph nodes (*r*_spearman_ = 0.498, *P* = 0.037) metastasis. There was a strong and positive correlation between SUAL at the time of diagnosis and asymptomatic brain metastasis (*r*_spearman_ = 0.647, *P* = 0.0034) (Table 2).

**Table 2:** Correlation between study variables and the presence of brain metastasis

Variables	*r*_Spearman_^a^	*P* value^α^
**Gender**		
Male	0.304	0.196
Female	0.415	0.106
**Smoking habit**		
Absent	0.345	0.245
Present	0.401	0.096
**ECOG PS**		
0	0.305	0.259
1	0.345	0.107
2	**0.401**	**0.045**
**Number of metastatic sites**		
Single site	0.241	0.209
Multiple sites	**0.497**	**0.024**
**Region of metastasis**		
Lung	0.194	0.205
Pleura	0.207	0.346
Liver	0.345	0.194
Bone	**0.456**	**0.033**
Adrenal gland	0.241	0.174
Intraabdominal lymph nodes	0.178	0.245
Supraclavicular/cervical lymph nodes	**0.498**	**0.037**
Peritoneum	0.269	0.378
**SUAL higher than the cut-off value**^**b**^	**0.647**	**0.0034**

*Abbreviations*: *ECOG* Eastern Collaborative Oncology Group, *PS* performance status, *SUAL* serum uric acid level

^α^*P*: a *P* value less than 0.05 was considered statistically significant and shown in bold font. The correlation coefficient of the significant *P* value is also indicated in bold

Statistical definitions: ^a^*r*: since the data did not show the normal distribution in the groups the Spearman’s correlation coefficient (*r*) test was used to determine the correlation between study variables

^b^The cut-off value for SUAL was calculated as 6.2 mg/dL

Univariate and multivariate regression analysis revealed that a SUAL above 6.2 mg/dL was an independent factor in predicting asymptomatic brain metastasis at the time of diagnosis (OR 6.243, 95% CI 2.943–7.964; *P* = 0.033) (Table 3).

**Table 3:** Determination of the independent variables affecting asymptomatic brain metastasis at time of the diagnosis in patients with extensive-stage small cell lung cancer

Variables	Univariate analysis	Multivariate analysis			
	***P* value**	***P* value**	**OR**	**95% CI lower**	**95% CI upper**
**Gender** (female vs. male)	0.245				
**Smoking habits** (present vs. absent)	0.301				
**ECOG PS** (2 vs. 0 and 1)	0.194				
**Number of metastatic sites (**multiple vs. single site)	0.094				
**Metastasis site at the time of the diagnosis (**bone vs. other sites)	**0.031**	0.129	1.818	0.135	3.422
**SUAL** (≥ cut-off value vs. < cut-off value)^a^	**0.005**	**0.009**	**3.446**	**1.337**	**5.480**

Statistical definitions: the variables in the table were included in the model because they were found to be significant in the univariate analysis. The variables that were not included in the model were not significant in the univariate analysis. Multivariate (binary) logistic regression analysis was performed because the data were not equally distributed. A *P* value less than 0.05 was considered statistically significant and shown in bold font. While the probability of predicting asymptomatic brain metastasis at time of diagnosis was 88.7% at the beginning, this rate increased to 94.6% with the model created. The *P* value for the Omnibus test for the adequacy of the model was calculated as 0.05. The Nagelkerke R squared value was 0.501

*Abbreviations*: *OR* odds ratio, *CI* confidence interval, *ECOG* Eastern Collaborative Oncology Group, *PS* performance status, *SUAL* serum uric acid level

^a^The cut-off value for SUAL was calculated as 6.2 mg/dL

## Discussion

A risk factor predicting when brain metastases will develop in SCLC patients is not clear. In this study, a SUAL above 6.2 mg/dL was identified as an independent factor for asymptomatic brain metastasis at diagnosis in patients with advanced SCLC. This is the first research that we know of to determine the relationship between asymptomatic brain metastasis and SUAL.

Despite the current rapid progress in molecular oncology and treatment options, platinum and etoposide are still the most important treatment strategies in small cell lung cancer [2, 3]. In recent studies, combining standard platinum and etoposide treatment regimens with immune checkpoint inhibitor monoclonal antibodies has resulted in a significant increase in treatment response and survival [2–4]. Although it is an aggressive type of cancer, it is clinically important that it is highly sensitive to chemotherapy and radiotherapy [2, 4]. Therefore, it is recommended that brain imaging be requested in all patients diagnosed with limited or diffuse disease, even if they are asymptomatic.

Uric acid, which is one of the end products of nucleotide metabolism, is one of the end products of nucleotide metabolism. Studies investigating the relationship between SUAL and cancer in literature focused on the proinflammatory effect of uric acid [5, 6, 15]. On the other hand, it has been stated that uric acid in high concentrations prevents lipid per-oxidation, scavenges free radicals, and may have anti-cancer effects. There are studies on the association of increased serum uric acid levels with cancer development and prognosis in a patient with cancer [16].

The studies suggest that SUAL may be an independent prognostic factor in patients with cancer is common. In recent years, there has been an increase in the number of studies attempting to show the prognostic and predictive value of SUAL in different types of cancer. It has been determined that SUAL has been investigated as a prognostic factor alone or in combination with other molecules in various cancer types. Research on lung cancer is also among these studies [17–19]. There are limited studies investigating the prognostic value of SUAL in SCLC. In a study by Wang et al. [19], it was determined that the tumor burden is higher, and the prognosis is poor in patients with high SUAL. On the other hand, there is no study investigating the relationship between asymptomatic brain metastasis and SUAL in small cell lung cancer.

However, in the results of the first known study investigating the prognostic value of SUAL in non-small cell lung cancer, it was reported that the rate of brain metastasis was higher and the time to brain metastasis was shorter in patients with SUAL above 7.49 mg/dL [17].

One of the main limitations of our study is the relatively low number of patients. Secondly, the fact that it is a study based on retrospective data can be considered an important limitation. Although people with gout or gout attacks were excluded from the study, the lack of data on nutritional characteristics can be considered a limitation. In particular, the fact that the patients’ alcohol and red meat consumption in the recent days before giving samples for blood tests is unknown may pose a question mark for SUAL. No anesthetic was used before and during blood collection. Considering the predictions about the effect of stress on serum uric acid levels, the fact that blood samples were taken calmly after resting in the laboratory center reserved for oncology patients where routine blood samples are taken may suggest that possible stress-related blood level changes for uric acid may have been relatively prevented. Therefore, there is a need for studies on a large number of patients in which the predictive value of SUAL can be determined by measurements made within a standard diet and timing. However, despite all these limitations, predicting brain metastasis in asymptomatic patients, regardless of stage, with an easily applicable and accessible biomarker is important for patient management.

## Conclusions

It can be thought that the predictive and prognostic value of SUAL, which is an easily accessible and measurable blood biomarker, may be important in predicting the development of brain metastasis in SCLC patients in clinical practice. It is recommended to develop this research with a larger patient series and to establish a nomogram. In conclusion, a serum uric acid level of 6.2 mg/dL and above at the time of diagnosis may predict asymptomatic brain metastasis in patients with SCLC.

## Data Availability

No, all the materials are owned by the authors and/or no permissions are required.

## Abbreviations

CI: Confidence interval
ECOG: Eastern Collaborative Oncology Group
Min–max.: Minimum–maximum
OR: Odds ratio
PS: Performance status
SUAL: Serum uric acid level
SCLC: Small cell lung cancer
